# Differential Effects of Adolescent Fatty Acid Amide Hydrolase and Monoacylglycerol Lipase Inhibition on Adolescent and Adult Behaviors in Male Rats

**DOI:** 10.21203/rs.3.rs-7512305/v1

**Published:** 2025-09-18

**Authors:** Mary Estes, Vishnu Guda, Emei Thompson, Daniela Rodrigues de Oliveira, David Nowak, Cecilia Hillard, John R. Mantsch

**Affiliations:** Medical College of Wisconsin

**Keywords:** 2-AG, anandamide, MAG lipase, FAAH, adolescence, social, play, cocaine, anxiety

## Abstract

**Rationale:**

Endocannabinoid signaling during adolescence plays a critical role in brain development and shapes both healthy and maladaptive behaviors, influencing the risk of neuropsychiatric disorders in adulthood.

**Objectives:**

The present study examines the effects of disrupted catabolism of the endocannabinoids 2-arachidonylglycerol (2-AG) and *N*-arachidonoylethanolamine (AEA) during early adolescence on adolescent and adult behaviors in male rats.

**Methods:**

Male Sprague-Dawley rats received daily injections with the monoacylglycerol lipase inhibitor JZL184 (JZL; 10 mg/kg, ip), the fatty acid amide hydrolase inhibitor URB597 (URB; 0.4 mg/kg, ip) or vehicle (saline) for ten days during early adolescence (PND31–40) and were tested for play behaviors and behavior on the elevated plus maze in adolescence and social preference, open field behavior and cocaine self-administration and seeking in adulthood.

**Results:**

Administration of JZL during adolescence increased interactive but not solo play or exploratory behaviors. Adolescent administration of JZL increased cocaine self-administration under a progressive ratio schedule of reinforcement and cocaine seeking, without effects on fixed-ratio cocaine self-administration or cocaine-primed reinstatement during adulthood, suggesting that elevated levels of 2-AG during adolescence may increase the risk for adult substance use disorders. Adolescent administration of URB promoted social choice during adulthood, consistent with a role for adolescent AEA in shaping social behavior during adulthood. Neither drug altered anxiety-associated behaviors during adolescence or adulthood.

**Conclusions:**

These findings are consistent with a critical role of endocannabinoid signaling during adolescence in shaping long-term behavioral outcomes, including vulnerability to addiction and social functioning in adulthood.

## Introduction

Adolescence is a critical period of development marked by heightened neural plasticity and profound changes in cognition, social behaviors, and emotional regulation. The adolescent brain is highly sensitive to environmental influences, including stress, substance use, and social context, which can shape trajectories of healthy or maladaptive behavior well into adulthood. Understanding the neurobiological processes that influence brain development during this time is essential for identifying risk factors for mental health disorders and for informing targeted interventions in adolescent populations.

The endocannabinoid system plays a key role in adolescent development. This system includes cannabinoid type receptors (CB1 and CB2), endocannabinoids (eCBs), and the enzymes responsible for their biosynthesis and degradation (Hillard 2015). The two most studied eCBs are 2-arachidonylglycerol (2-AG) and N-arachidonoylethanolamine (anandamide or AEA). Monoacylglycerol lipase (MAGL) and fatty acid amide hydrolase (FAAH) are the primary enzymes responsible for the degradation of 2-AG and AEA, respectively. The conFigureuration of the endocannabinoid (eCB) system is unique during adolescence, characterized by distinct patterns of CB1 receptor expression, FAAH and MAGL activity, and fluctuating levels of 2-AG and AEA (Ellgren et al. 2007; Heng et al. 2011; Lee et al. 2013; Long et al. 2012), rendering adolescents particularly vulnerable to perturbations in eCB signaling.

Rodent studies have demonstrated that, during early to mid-adolescence (PND35–45), eCBs regulate synaptic inputs into the prefrontal cortex, facilitating disinhibition of cortical network function (Cass et al. 2014; Molla et al. 2024). Notably, maintaining elevated adolescent-like eCB signaling into adulthood either in rats carrying a gain-of-function mutation in CB1 receptors (Schneider et al. 2015) or in mice harboring a human FAAH C385A polymorphism that reduces enzyme function (Burgdorf et al. 2020; Gee et al. 2016) - preserves adolescent behavioral phenotypes. These findings support the broader role for adolescent eCB signaling in shaping adult anxiety-related (Molla et al. 2024) and social behaviors (Alteba et al. 2020)), cognition (Renard et al. 2013), stress responses (Lee et al. 2015), and sensitivity to drugs of abuse (Pistis et al. 2004). In humans, altered eCB signaling during adolescence has similarly been linked to various neuropsychiatric conditions (Tansey et al. 2025), including depression (Jaster et al. 2025), anxiety disorders (Marusak et al. 2025), autism spectrum disorder (Aran et al. 2019) and post-traumatic stress disorder (Marusak et al. 2024).

In the present study, we examined the effects of MAG lipase or FAAH inhibition during early adolescence (PND31–40) on adolescent and adult behaviors in male rats using daily administration of the MAG lipase inhibitor, JZL184 (JZL; (Long et al. 2009; Pan et al. 2009) or the FAAH inhibitor, URB597 (URB) (Fegley et al. 2005), respectively. While the objective is to determine the effects of elevated 2-AG and AEA during adolescence, it is important to recognize that FAAH hydrolyzes a number of other fatty acid amides including oleoylethanolamide (OEA) and palmitoylethanolamide (PEA), both of which are biologically active, while MAG lipase breaks down other monoacylglycerols. Specifically, we tested rats for play and elevated plus maze behavior during adolescence and social choice, open field behavior, and cocaine self-administration/seeking during adulthood. Altogether our findings demonstrate the adolescent JZL and URB administration produce divergent effects on behavior that persist into adulthood.

## Methods

### Subjects

Male Sprague Dawley rats were purchased from Envigo/Inotiv and arrived on postnatal day 21 (PND 21). Rats were pair-housed upon arrival and maintained on a 12-hour light/12-hour dark cycle in a humidity- and temperature-controlled facility. Food and water were provided *ad libitum*. All experiments were approved by the Medical College of Wisconsin IACUC and conducted in accordance with NIH guidelines.

### Drugs

JZL184 (10 mg/kg) and URB597 (0.4 mg/kg) (Cayman Chemicals, MI) were administered using a 1:1:8 EtOH:kolliphor:saline vehicle. Controls were administered vehicle injections only. Doses were chosen based on known behavioral changes in socialization and anxiety-related behaviors (Aliczki et al. 2013; Alteba et al. 2020; Fidelman et al. 2018; Sciolino et al. 2011). Cocaine HCl was obtained through the NIDA Drug Supply Program. The cocaine was dissolved in saline.

### Adolescent drug treatment

Adolescent treatment regimens consisted of daily intraperitoneal (ip) injections of either JZL184 (10mg/kg), URB597 (0.4mg/kg) or vehicle from PND31–40. Starting on PND 28, the animals were weighed and handled daily.

### Behavioral Testing

**Figure 1.**
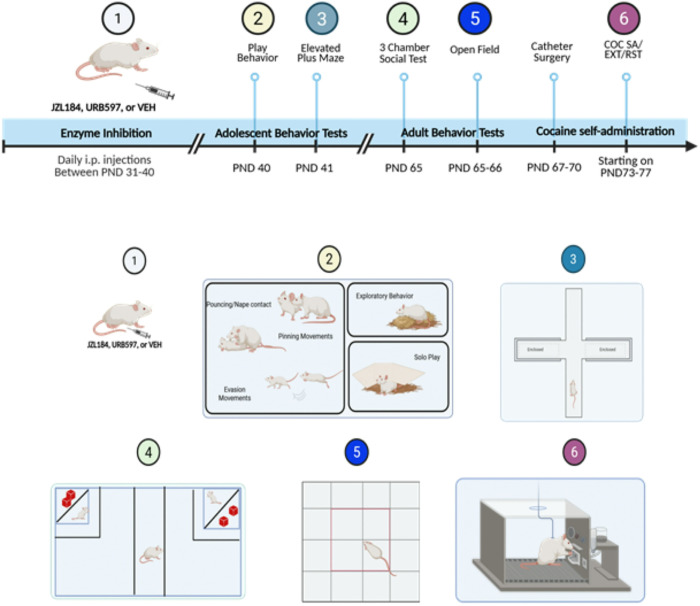
Overview of experimental design. Rats were treated with the monoacylglycerol lipase inhibitor JZL184, the fatty acid amide hydrolase inhibitor URB597, or vehicle (VEH) from post-natal day (PND) 31 through PND41 prior to testing for play behavior and behavior on the elevated plus maze during adolescence and behavior in the 3-chamber social choice test and in the open field during adulthood. Subsequently rats received venous catheters and were tested for cocaine self-administration (COC SA), cocaine seeking/extinction (EXT) and cocaine primed reinstatement (RST). Created with BioRender.com.

**Figure 2.**
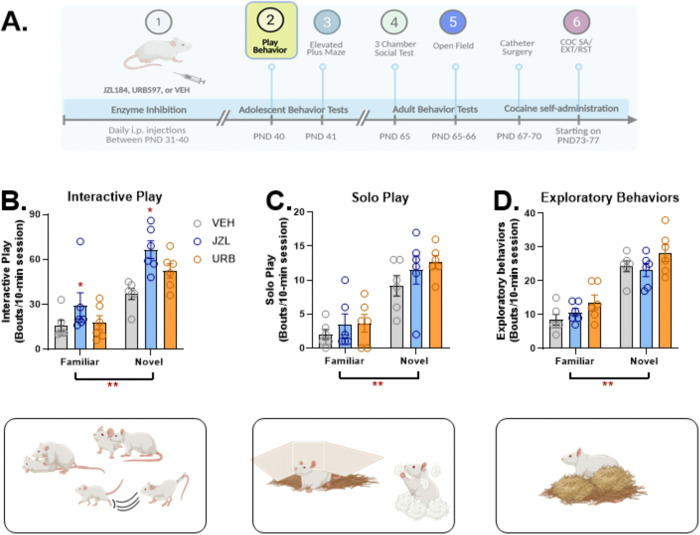
Effects of adolescent JZL184, URB597, and vehicle administration on adolescent play behavior. Rats were tested for play behavior in a familiar (n=6/group) or novel (n=6/group) context on PND40, two hours after the final injection (2A). Data represent interactive (physical play) (2B), solo play (2C), and exploratory behaviors (2D) measured over a 10-min period in rats treated with JZL184 (JZL), URB597 (URB), or vehicle (VEH) during adolescence. Overall, each behavior was increased when tested in a novel versus a familiar context (**p<0.001 for each comparison). Overall, time spent engaged in interactive play was significantly increased in JZL-treated rats relative to vehicle controls (*p=0.002; Tukey’s HSD). Created in part with BioRender.com.

**Figure 3.**
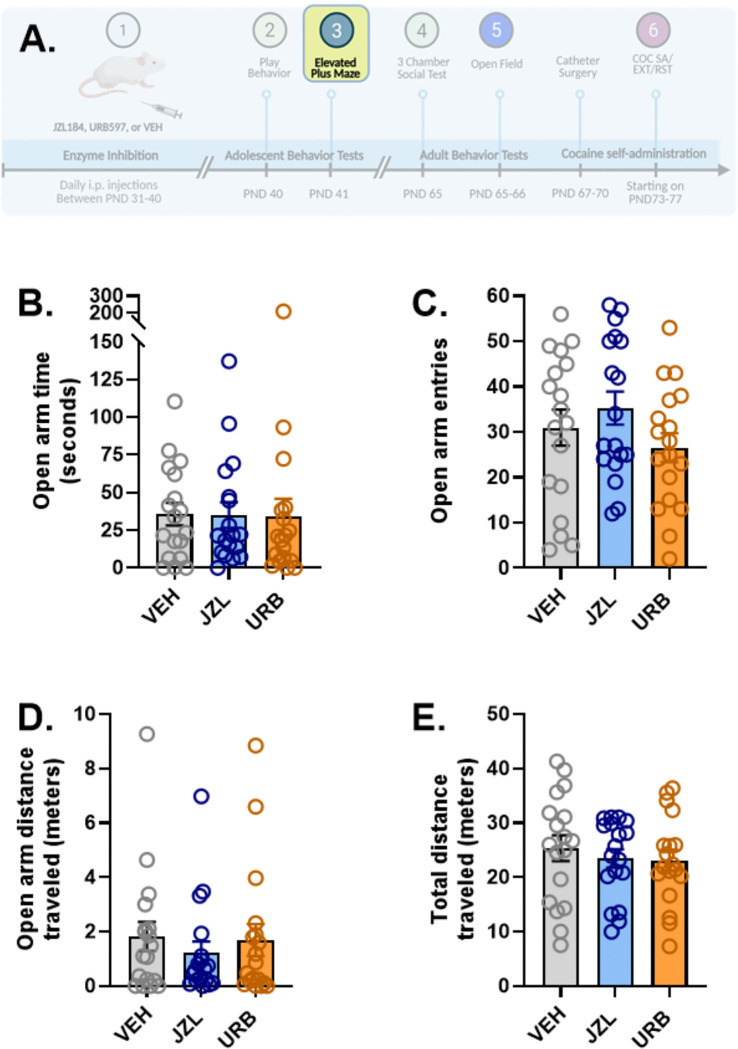
Effects of adolescent JZL184, URB597, and vehicle administration on behavior on the elevated plus maze in adolescent rats. Rats (n=18/treatment group) were tested for behavior on the elevated plus maze (EPM) on PND41 (3A). No effects of adolescent drug treatment were observed on open arm time (3B), open arm entries (3C), distance traveled on the open arms (3D), or total distance traveled on the EPM (3E). Created in part with BioRender.com.

**Figure 4.**
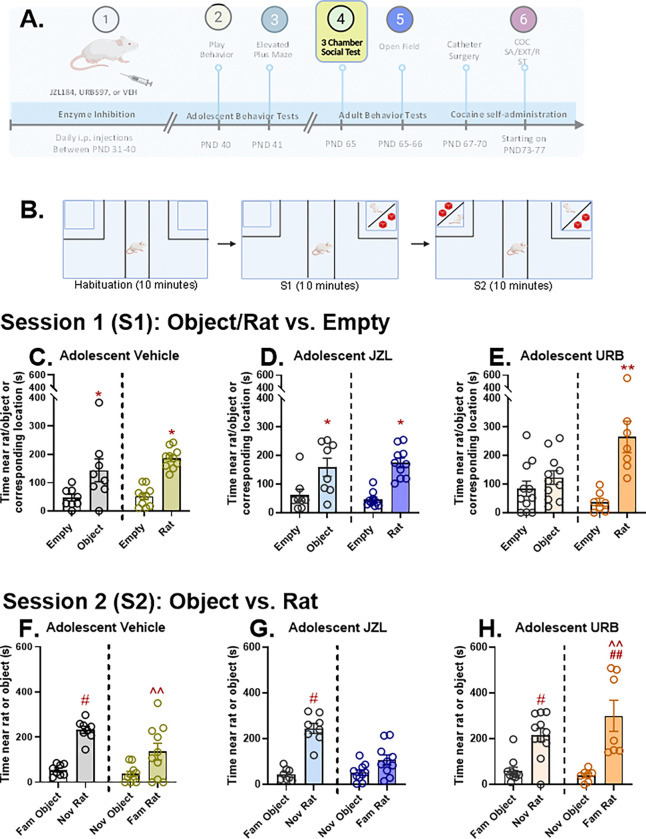
Effects of adolescent JZL184, URB597, and vehicle administration on behavior on social preference in adult rats. Rats were tested for social preference using the 3-chamber social test on PND65 (4A). Testing consisted of three phases (4B): a 10-min habituation phase, a 10-min phase where rats chose between a novel rat or object and an empty compartment (S1), and a 10-min phase where rats chose between a novel object or rat and familiar rat or object (S2). Data represent the time spent in close proximity to an object, rat, or a corresponding location in an empty chamber. During the S1 phase, rats treated with vehicle (4C) or JZL (4D) during adolescence showed comparable preference for a novel rat or novel object over the empty compartment (veh/rat, n=8; veh/object, n=10; JZL/rat, n=8; JZL/object=n=10; Tukey’s HSD test; p <0.05). By contrast, rats treated with URB (4E) did not display a preference for a novel object over and empty compartment (n=12) but showed very strong preference for a novel rat (n=7) that was greater than that observed in VEH or JZL rat (**p=0.0001, rat vs. empty and p=0.0006 rat vs. object; Tukey’s HSD). During the S2 phase, a significant adolescent drug treatment × prior rat/object exposure × preference interaction was found (F_2,48_ = 4.168, p=0.021). Time spent near the rat or object during the session in rat that received adolescent vehicle (4F), JZL (4G), or URB (4H) is shown. In all cases, rats preferred a novel rat over a familiar object (^#^p<0.0001; Tukey’s HSD). Rats treated with vehicle or URB, but not JZL, preferred a familiar rat over a novel object (^^^^p<0.05; Tukey’s HSD). Rats treated with URB during adolescence spent more time with a familiar rat than vehicle or JZL-treated rats (^##^p<0.001; Tukey’s HSD). Created in part with BioRender.com.

**Figure 5.**
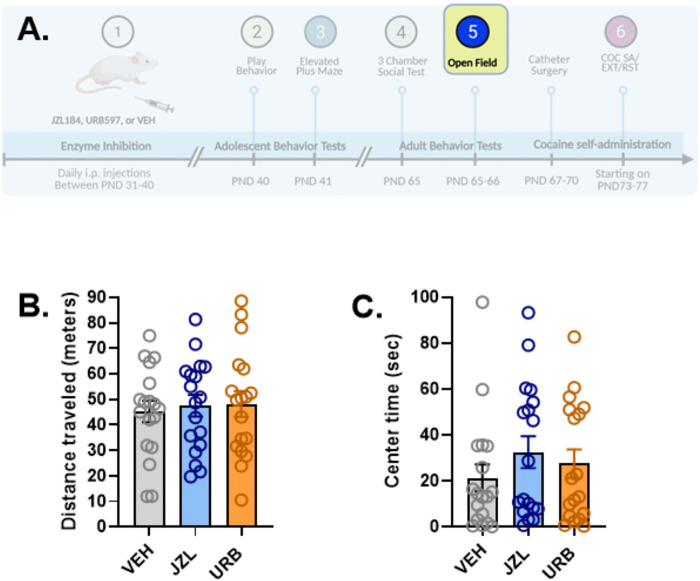
Effects of adolescent JZL184, URB597, and vehicle administration on open field behavior in adolescent rats. The effects of adolescent drug treatments (n=18/treatment group) on open field behavior (20-min sessions) were tested on PND65 (5A). No differences among treatment groups were observed for either the total distance traveled (5B) or the time spent in the center of (5C) the open field chamber. Created in part with BioRender.com.

**Figure 6.**
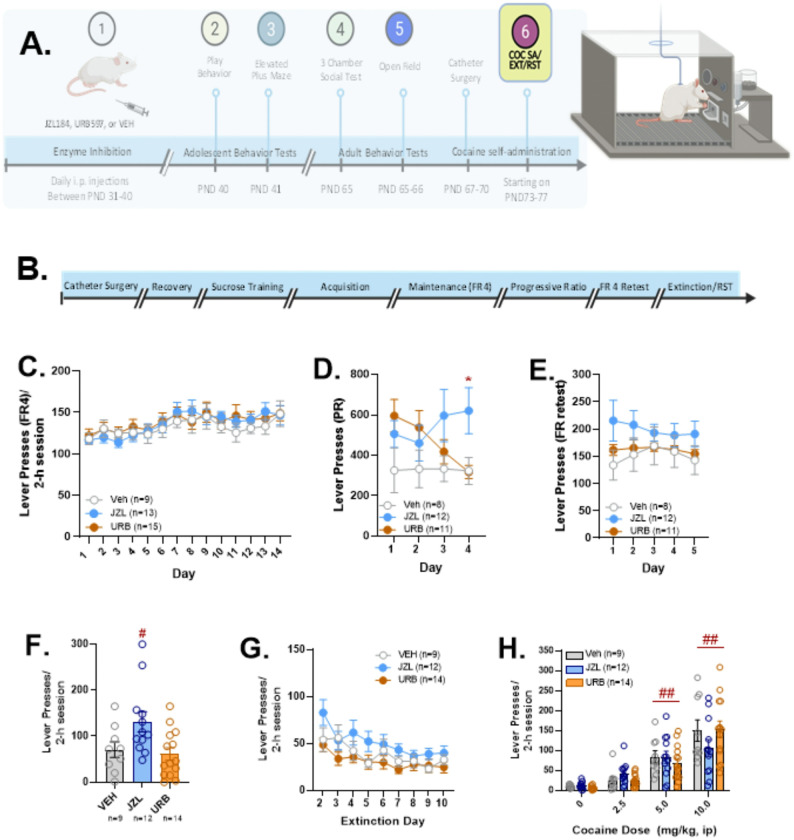
Effects of adolescent JZL184, URB597, and vehicle administration on cocaine self-administration, seeking, and reinstatement in adult rats. Rats were implanted with venous catheters and tested for cocaine self-administration, seeking, and cocaine-primed reinstatement during adulthood (6A), the time-course for surgery, recovery, training, and testing is shown in 6B. Responding across 14 days of fixed ratio 4 (FR4) self-administration did not differ across adolescent treatment groups (6C). When rats were tested under progressive ratio (PR) conditions (6D), a significant treatment × SA day interaction was found (F_6,32_= 3.482; p = 0.009). On the final day of testing, PR responding was significantly increased in JZL184 (JZL) treated rats relative to URB597 (URB) treated rats or vehicle (VEH) controls (*p≤0.05; Tukey’s HSD). When rats were subsequently retested for FR testing over a 5-day period (6E), statistically significant differences were once again no longer observed. When rats were tested for seeking (i.e., on extinction day one; 6F), a significant effect of drug treatment was found (F_2, 32_=4.785, p=.015), Responding in JZL rats was significantly increased relative to URB rats (^#^p=.016; Tukey’s HSD) but not VEH controls (p=.077). Differences were not found during extinction days 2–9 (6G). When rats were tested for cocaine-primed reinstatement (6E) an overall effect of dose was observed (F^3,42^=51.39; p<0.001). Overall, reinstatement was observed at 5 and 10 mg/kg cocaine doses (^##^Tukey’s HSD; p<0.001 vs. saline) but did not differ across adolescent treatment groups. Sample sizes varied across testing due to removal of rats based on catheter patency. Created in part with BioRender.com.

To evaluate the short- and long-term effects of increased eCB levels, we conducted a series of tests assessing social, anxiety-related, and drug self-administration/seeking behaviors (Figure 1). In adolescence, we measured social play behavior (PND40) (Figure 2A) and used the elevated plus maze to assess anxiety-related behaviors (PND41) (Figure 3A). After the elevated plus maze testing, the animals remained in the colony, where they were weighed and handled daily. In adulthood, we used the three-chamber social test (PND65) to evaluate social behaviors (Figure 4A) and the open field test to assess anxiety-related behaviors (PND66–67) (Figure 5A) prior to surgically implanting rats with venous catheters and testing for cocaine self-administration and seeking (Figure 6A).

### Play Behaviors

Two hours after the final injection (PND 40), play behavior was recorded during the dark cycle using infrared cameras. Rats were either recorded in a familiar (home cage) or novel cage environment. Each cage (36.5 cm × 25.5cm × 18.5 cm) was furnished with Santi-Chip bedding and 2 paper towels. Behavior was scored by two observers for interactive/physical play (pouncing/nape contact, pinning, and evasion), solo play (hiding in/under paper towel, playful movements when alone in area of cage), and total time of play (Himmler et al. 2013; VanRyzin et al. 2020). Additionally, exploratory behaviors such as rooting, rearing or rooting through bedding were recorded.

### Elevated Plus Maze (EPM)

EPM (Stoelting 60240) testing was conducted 24 hours after the final injection (PND41). Animals were transferred to the testing room and allowed to habituate for 30 minutes. The plus-shaped maze consisted of 2 open arms, 2 enclosed arms, and a center zone where the arms met (arm length = 50 cm, wall height = 40 cm). The EPM was elevated at a height of 50 cm from the floor. An overhead camera (info) was positioned cm above the floor. At the start of the 10-minute test, animals were placed in the center zone facing an open arm. A luxmeter was used to ensure the environment was between 40–55 lux. Recorded videos were analyzed by ANY-maze (Stoelting, V. 7.4). Variables analyzed included time spent on the open arms, distance traveled on open arms, time spent in the closed arms, distance traveled on the closed arms, entries to the open arms, and total distance traveled.

### 3-Chamber Social Test

The three-chamber social test evaluates social behaviors by assessing the preference between a social environment and a non-social environment. The apparatus was a three-chambered, rectangular box. The dimensions of center/start zone were 19 cm L × 20 cm W × 28 cm H and the two end compartments were 51 cm × 61 cm × 32 cm. The testing was separated into three 10-minute phases: habituation, S1, and S2. At the start of habituation, rats were placed in the center zone and allowed to explore all areas. Between phases, rats were placed back in the start zone without access to the side zones. Prior to starting S1 phase, either an unfamiliar rat or an unfamiliar object (a 3D printed red block) was placed into zone 1 (the end compartment on the right side of apparatus). Half of the rats in each treatment group were tested with a rat during this phase; the other half were tested with an the red block. During the 10-min preference test session, rats had access to all of the chambers. After the S1 session, rats were once again contained in the center zone. For testing during the 10-min S2 phase, either a novel object (for rats initially tested with a rat) or a novel rat (for rats initially tested with an object) was placed into zone 1 so that the choice was for a novel object and familiar rat in half of the subjects and between a novel rat and a familiar object in the remaining subjects. Measures recorded included the distance traveled and time spent in each zone/compartment and time spent engaging the object/rat or in the corresponding location in the empty compartment during the 10-minute sessions.

### Open Field

The open field test was conducted to evaluate the long-term effects of manipulating eCB levels on anxiety-related behaviors. The test consisted of two 20-minute trials performed on consecutive days. Each trial was recorded and analyzed using ANY-maze software. The open field apparatus was a black, opaque square measuring 91.5 cm L × 91.5 cm W × 46 cm H. The square area was divided into a center zone and a perimeter zone, with the center zone measuring 46 × 46 cm. Animals were transported to the testing room and allowed to habituate for 30 minutes before testing. The animal was then placed into the center of the open field to begin the trial. The apparatus was cleaned with 70% isopropyl alcohol between tests. Variables recorded included total distance traveled and time spent in the center zone. To evaluate behavioral changes between days, variables analyzed included habituation scores for total distance traveled, time spent in the center zone, and entries to the center zone.

### Surgery

Between PND68–70, rats were implanted with venous catheters for drug self-administration under isoflurane anesthesia, as previously described (McReynolds et al. 2016). Animals received the antibiotic cefazolin (2 mg/kg, iv) for 5 days, and the nonsteroidal anti-inflammatory drug (NSAID) meloxicam (2 mg/kg, subcutaneous) for 3 days following surgeries. Rats recovered for 7 days before beginning cocaine self-administration.

### Cocaine Self-Administration

Self-administration was conducted in operant conditioning chambers with retractable levers and stimulus lights above each lever (Med Associates, Inc.). Rats were food restricted (20g/day) 24 hours before the first session and remained food deprived until the rat met the criterion for acquisition. Animals were initially trained to press a lever under a continuous reinforcement schedule to receive sucrose pellets until they received ≥90 pellets for two consecutive sessions. Thereafter, rats were provided an opportunity to intravenously self-administer cocaine (0.5 mg/kg/200μl, iv) by pressing the same lever, cued by a stimulus light during daily 2-h sessions. After each 5-sec infusion, there was a 10-second timeout period during which the stimulus light was turned off and lever presses were recorded but not reinforced. Responding on a second inactive lever was also recorded. Self-administration training was initially conducted under a fixed ratio one (FR1) schedule of reinforcement and the schedule requirement was incrementally increased to FR4. Once stable responding was observed under the FR4 schedule (two consecutive days with <15% variability in cocaine infusions), rats entered the maintenance phase of testing, during which cocaine was available under the FR4 schedule of reinforcement during 2-h sessions for 14 consecutive days. After 14 days, rats were tested for self-administration under a progressive ratio (PR) schedule for four consecutive days as previously described (Mantsch et al. 2010). Under this schedule the response requirement for cocaine delivery was increased with successive infusions using the following progression: 1, 2, 4, 6, 9, 12, 15, 20, 25, 32, 40, 50, 62, 77, 95, 118, 145, 178, 219, 268, 328, 402, 492, 603, 737, 901, 1102, 1347, 1646, 2012. The progression continued until the rat failed to complete a ratio requirement within a 1-h period. Following PR testing, rats were once again returned to the FR4 schedule of cocaine reinforcement for five consecutive days. In all cases, cocaine infusions and responses on the cocaine-reinforced lever were recorded and reported. Following the 5-day FR self-administration period, cocaine access was removed and testing for cocaine seeking, extinction, and cocaine-primed reinstatement was conducted.

### Extinction and Reinstatement

Extinction training was conducted until a rat pressed the lever previously reinforced by cocaine less than 20 times for two consecutive sessions. Conditions during the 2-h extinction conditions were identical to those during FR self-administration except no drug was delivered and rats were untethered during the sessions. Responding on day one of extinction was also used as a measure of cocaine seeking. Once the extinction criterion was met, reinstatement testing was initiated. Rats were tested for reinstatement across a range of cocaine doses (0, 2.5, 5, and 10 mg/kg, ip). Cocaine was administered immediately prior to placing rats into the operant conditioning chambers. The conditions during the 2-h reinstatement sessions were otherwise identical to those during extinction. Each rat was tested for reinstatement in response to each cocaine priming dose in counterbalanced sequence. Rats were required to display extinction-level cocaine seeking (<20 responses) during at least two extinction sessions between reinstatement tests. For all sessions, cocaine (active) and inactive lever responding were recorded.

### Statistical Analyses

Power analysis was conducted using G*Power. Statistical analyses were completed using SPSS statistics software (IBM, version29). Data were analyzed with one-way ANOVAs and one- or two-way repeated measures ANOVAs. All ANOVAs were followed, when appropriate, with post hoc testing using Tukey’s HSD tests. Statistical significance was defined as p < 0.05.

## Results

A schematic depicting the experimental timeline is included in Figure 1. Male rats (n=18/group) received daily injections (at 0900h) with the MAGL inhibitor JZL184 (10 mg/kg, ip), the FAAH inhibitor URB597 (0.4 mg/kg, ip) or vehicle (VEH; 1:1:8 EtOH:kolliphor:saline) for ten days during early adolescence (PND31–40). Drug treatments had no effects on body weight during adolescence or adulthood (Table 1).

### Adolescent Play Behaviors

On PND40, two hours following the last injection, 12 rats from each treatment group were tested for play behaviors during a 10-min window in either a familiar (home cage; n=6/group) or novel (fresh but otherwise identical cage; n=6/group) environment. The effects of adolescent JZL, URB, or vehicle on play behaviors are shown in Figure 2. Behaviors were categorized as interactive/physical play (Figure 2B), solo play (Figure 2C), and exploratory (Figure 2D) (see Materials and Methods). 2-way (treatment × test environment) ANOVAs were used to assess the effects of adolescent drug treatments. Each behavior was increased when measured in a novel vs. familiar environment (overall effects of environment: F_1,30_=47.40, p<0.001 for interactive/physical play; F_1,30_=85.911, p<0.001 for solo play; F_1,30_=48.601, p<0.001 for exploratory behavior). Total play time was also increased in the novel environment (F_1,30_=28.207, p<0.001; *Figure S1*). Overall, treatment effects on interactive/physical play (F_2,30_=7.653, p = 0.002) and exploratory behavior (F_2,30_=3.525, p = 0.04) but not solo play or total play time were observed. Post-hoc testing showed that JZL treatment significantly increased physical/interactive play compared to vehicle controls in both environments (Tukey’s HSD; p=0.002). Although there was a trend towards increased interactive play in rats treated with URB relative to vehicle controls when tested in the novel environment, this difference was not statistically significant (planned comparison; p=0.095). Significant differences across treatment groups were not observed for exploratory behavior.

### Adolescent Elevated Plus Maze Behavior

Behavior on the elevated plus maze was measured on PND41 in all rats (n=18/group) and is shown in Figure 3. No significant effects of adolescent drug treatment were found on any of the following measures: open arm time (Figure 3B), open arm entries (Figure 3C), open arm distance travelled (Figure 3D), closed arm time (*Figure S2A*), closed arm distance travelled (*Figure S2B*), closed arm entries (*Figure S2C*), or total distance traveled (Figure 3E) (one-way ANOVAs).

### Adult Social Preference

On PND65, rats underwent social preference testing using a 3-chamber apparatus (Figure 4; *Figure S4*). During consecutive 10-min sessions (Figure 4B), rats were tested for 1) baseline preference (*Figure S3*); 2) preference for a novel object or an untreated novel peer rat vs. an empty chamber; and 3) preference for an object vs. a rat. Under baseline conditions, there was no overall preference or within-group side preferences based on the times spent *(Figure S3A)* and distances travelled (*Figure S3B*) in each compartment. Moreover, neither time spent (*Figure S3A*) nor distance traveled (*Figure S3B*) in each compartment differed across treatment groups under baseline conditions.

During the second 10-min session rats in each group were tested for their preference for a compartment containing either a novel object or a novel rat relative to an empty compartment. We initially conducted a 3-way ANOVA examining preference for the occupied vs. empty compartment (repeated measure), the effect of a novel rat vs. novel object in the occupied compartment, and the influence of adolescent drug treatment. We assessed three measures: 1. Time spent in the occupied vs. empty compartment *(Figure S4A-C)*, 2. Distance traveled in the occupied vs. empty compartment (*Figure S4G-I)*, and 3. Time spent in the immediate proximity of the novel object or rat vs. the corresponding location in the empty compartment (near time; Figure 4C–E). Each measure indicated an overall preference for the occupied chamber over the empty chamber (Time spent: F_1,48_=34.687, p<0.001; Distance traveled: Time spent: F_1,48_=57.398, p<0.001; Near time: F_1,48_=57.498, p<0.001). Overall, no effects on adolescent drug treatment were found. Interactions between compartment preference and what resided in the occupied compartment (rat vs. object) were found for both distance traveled (F_1,48_=9.047, p=0.004; *Figure S4G-I*) and time spent near the object/rat or corresponding empty compartment location (F_1,48_=7.296, p=0.01). In each case the behavior suggested overall greater preference for a novel rat or a novel object over the empty compartment (Tukey’s HSD test; p <0.05). While we did not observe statistically significant adolescent treatment × compartment preference × object/rat interactions, a trend towards a statistically significant interaction was found for the near time measure (p=0.088). To more closely examine this, we analyzed the relative preferences (near times) for a novel rat vs. a novel object in each adolescent treatment group. While the preference for a rat vs. an object was comparable in rats that received vehicle or JZL during adolescence (main effects of preference: F_1,9_=30.04, p=0.0004 for vehicle and F_1,9_=24.54, p=0.0008 for JZL; no interaction between preference and rat/object), URB-treated rats showed high preference for a novel rat but no preference for a novel object. A 2-way empty vs. occupied compartment × rat vs. object ANOVA showed an overall preference effect (F_1,32_=17.79, p=0.0002) and a preference × occupant (rat/object) interaction (F_1,32_=8.897, p=0.0005). Preference for the rat-occupied chamber (Tukey’s HSD; p =0.0001) but not the object-occupied chamber over the empty chamber (p=0.756) was observed, with greater preference displayed for a novel rat versus a novel object (p=0.0006), indicating that URB-treated rats showed high social preference but were indifferent to a novel object.

During the third 10-min session, rats were tested for their preference for a rat vs. an object. Notably, some of the rats had prior experience with the same object during the previous 10-min session, while the remainder of the rats had prior experience with the same rat. We initially conducted a 3-way ANOVA examining preference for the object- vs. rat-occupied compartment (repeated measure), the effect of exposure to the rat or object during the previous 10-min session, and the influence of adolescent drug treatment. Overall, rats preferred another rat over an object when given a choice as indicated by overall preference effects based on time spent (F_1,48_ = 37.448, p<0.001) and distance traveled (F_1,48_ = 241.896, p<0.001) in each compartment and the time spent in close proximity of the rat/object (near time; (F_1,48_ = 92.796, p<0.001). As expected, overall preference varied based on prior exposure to the rat or object. In particular, rats previously exposed to the rat showed less overall distance traveled (F_1,48_ = 11.572, p=0.001) during the session. This is likely the result of familiarity resulting from earlier exposure to the rat, as indicated by a significant preference × prior exposure effect (F_1,48_ = 65.648, p=0.001). Post-hoc testing revealed a significant reduction in distance traveled within the rat-containing compartment in animals previously exposed to the rat relative to animals previously exposed to the object (Tukey’s HSD; p<0.001). Interestingly, this effect varied depending on adolescent drug treatment as indicated by a significant overall effect of adolescent drug treatment on time spent in close proximity to the rat/object (near time; F_2,48_ = 4.168, p=0.021) and a significant adolescent drug treatment × prior rat/object exposure × preference interaction (F_2,48_ = 4.168, p=0.021). Post-hoc testing revealed that rats treated with URB during adolescence spent more time re-engaging a familiar rat compared to vehicle or JZL-treated rats (Tukey’s HSD; p<0.001 for each comparison). Indeed, while rats in each treatment group that were exposed to the object during the prior session showed preference for a novel rat (Tukey’s HSD; p<0.001 for each comparison), preference for the familiar rat over the novel object was most pronounced in URB treated-rats (Tukey’s HSD; p<0.0001) and was evident in vehicle treated-rats (p=0.004) but was not statistically significant in JZL-treated rats (p=0.102). Notably, preference for a familiar rat was lower than for a novel rat in animals treated with JZL (Tukey’s HSD; p=0.003) or vehicle (p=0.08) during adolescence but was significantly increased in URB-treated rats (p=0.031). Thus, adolescent URB treatment not only enhances social preference for a novel conspecific, but it promotes sustained engagement with a familiar conspecific.

### Adult Open Field Behavior

The effects of adolescent drug treatments on open field behavior during adulthood is shown in Figure 5. All rats (n=18 per treatment group) were tested for open field behavior during 20-minute sessions on PND65. No differences among treatment groups were observed for any of the open field measures (total distance traveled, Figure 5B; time spent in the center of the chamber, Figure 5C; or time spent freezing, *Figure S4C*; one-way ANOVAs). A subset of rats (n=12 per treatment group) was tested again on PND66. Rats habituated to the open field conditions as indicated by increases in the total distance traveled (2-way treatment × open field test day ANOVA; significant main effects of test day: F_1,11_=39.31, p<0.0001) and time spent in the center of the chamber (2-way treatment × open field test day ANOVA; significant main effects of test day: F_1,11_=10.85, p=0.007), and reductions in freezing (2-way treatment × open field test day ANOVA; significant main effects of test day: F_1,11_=72.15, p<0.0001). As was the case with day one of open field testing, an overall effect of adolescent drug treatment was not observed on the second day of testing, nor was a treatment × test day interaction.

### Adult Cocaine Self-Administration, Extinction, and Reinstatement

Cocaine self-administration (SA), extinction, and reinstatement in rats that received JZL, URB, or vehicle during adolescence are shown in Figure 6. Following open field testing, rats were implanted with venous catheters and provided access to cocaine for intravenous self-administration (Figure 6B). Due to issues related to catheter patency, only nine saline rats, 13 JZL rats, and 15 URB rats completed self-administration. There was no effect of adolescent drug treatment on the acquisition of cocaine SA as measured by the number of training days before rats met the acquisition criterion (Veh: 7.09 ± 0.53; JZL: 6.62 ± 0.27; URB: 6.60 ± 0.27). Following acquisition, rats underwent 14 days of self-administration under a fixed ratio 4 (FR4) schedule of reinforcement. Cocaine infusions and cocaine lever responses were recorded and analyzed using 2-way adolescent drug treatment × SA day ANOVAs. Overall, cocaine SA increased across the 14-day test period (F_13,182_ = 5.387, p<0.0001 for responses, Figure 6C; F_13,182_ = 5.834, p<0.0001 for infusions, *Figure S6A*). However, neither main effects of adolescent drug treatment nor treatment × SA day interactions were observed.

After 14 days of FR SA, the schedule was shifted from FR4 to progressive ratio (PR) for four days. Differences among adolescent treatment groups in cocaine-reinforced responses over the 4-day period were assessed using a 2-way treatment group × PR SA day (repeated measure) ANOVA. While no main effects of adolescent drug treatment or SA day on responses during PR testing were observed, there was a significant treatment × SA day interaction (F_6,32_= 3.482; p = 0.009, Figure 6D). Although no significant differences across treatment groups were observed on day one of testing, PR responding was elevated in rats that received URB or JZL relative to vehicle controls. However, PR responding in URB rats declined over time with a significant reduction on the final day of testing relative to day one (Tukey’s HSD test, p=0.004), suggesting that the initial increase may have reflected a deficit in behavioral flexibility rather than drug-motivation. On the final day of testing, PR responding was significantly increased in JZL-treated rats compared to either URB-treated rats (p=0.03) or vehicle controls (p=0.05), indicating that rats had heightened motivation for cocaine during adulthood following adolescent JZL administration. A 2-way treatment group × PR SA day ANOVA failed to show main effects of treatment condition or SA day on PR infusions or a significant interaction *(Figure S6B)*, although a trend towards a significant interaction was observed (F_6,38_ =2.141, p=0.07).

Following PR SA, rats were once again provided cocaine access under FR4 conditions for an additional 5 days. As was the case prior to PR SA, neither cocaine lever responses (Figure 6E) nor cocaine infusions (*Figure S6C)* differed across treatment groups and treatment group × SA day interactions were not observed (2-way treatment × day ANOVAs).

Next, rats underwent extinction training for ten days. As responding on the first day of extinction training can be used as an indicator of drug seeking, we analyzed day 1 responding independently from the other extinction days. One-way ANOVA revealed a significant effect of adolescent drug treatment (F_2, 32_=4.785, p=.015; Figure 6F). JZL-treated rats displayed higher cocaine seeking than URB-treated rats (p=.016) and a trend (p=.077) toward increased drug seeking compared to vehicle controls. By contrast, one-way ANOVA did not show significant differences in inactive lever pressing among adolescent treatment groups (*Figure S6D*). When we examined extinction over a subsequent 9-day period (Figure 6H), 2-way adolescent treatment × extinction day (repeated measure) ANOVA showed a significant overall extinction effect (F_9,252_=14.301; p<0.001) but no treatment effect or treatment × extinction day interaction.

Following extinction, rats were tested for cocaine-primed reinstatement of drug seeking across a range of cocaine doses (0, 2.5, 5, and 10 mg/kg, ip) in counterbalanced sequence (Figure 6H). A 2-way ANOVA revealed a significant main effect of cocaine dose (F_3,42_=51.39; p<0.001) but no effect of adolescent drug treatment and no dose × adolescent drug treatment interaction. Overall, cocaine dose-dependently reinstated extinguished cocaine seeking. Post-hoc testing revealed reinstatement at the 5 and 10 mg/kg, but not 2.5 mg/kg cocaine doses (Tukey’s HSD; p<0.001 vs. saline).

## Discussion

Adolescence is a critical developmental window during which life events can alter brain function to influence the trajectory of healthy and unhealthy behaviors across the lifespan. Adolescence also represents a key developmental period for the eCB system within which distinct patterns of CB1 receptors, FAAH, and MAGL expression and levels of 2-AG and AEA are observed, rendering adolescents uniquely susceptible to changes in eCB signaling (Ellgren et al. 2007; Heng et al. 2011; Lee et al. 2013; Long et al. 2012). Here we report that the MAGL inhibitor, JZL184, and the FAAH inhibitor, URB597, when administered during adolescence (PND31–40), produce divergent effects on behavior that persist into adulthood in male rats. Only JZL significantly increased adolescent play behavior. Adolescent administration of JZL but not URB heightened cocaine seeking behaviors during adulthood, suggesting that elevated 2-AG during adolescence may increase the risk for adult SUDs. Adolescent administration of URB but not JZL promoted social choice during adulthood, suggesting that AEA during adolescence is important for social development. Neither drug altered anxiety-associated behaviors related to risk assessment during adolescence or adulthood.

### Adolescent Play Behavior

Adolescent administration of JZL increased interactive but not solo play without altering general locomotor activity. There was also a non-significant trend toward an increase in URB-treated rats when they were tested in a novel environment. Adolescent social encounters in rats are associated with elevated brain levels of AEA but not 2-AG (Marco et al. 2011). Previous studies have demonstrated that acute URB administration promotes social play behavior (Trezza et al. 2012; Trezza and Vanderschuren 2008). Since we measured physical/interactive play behavior shortly after the final URB injection it is likely, based on the reported time course of eCB elevation following URB (Fegley et al. 2005) that brain AEA was increased at the time of testing. Thus, our observation that there was a statistically non-significant trend toward an increase in physical/interactive play behavior following repeated URB administration is consistent with these results. It is possible that the lack of statistically significance is the result of tolerance to the effects of URB with repeated administration. Based on the time course for JZL increases in brain 2-AG (Long et al. 2009), brain 2-AG concentrations were also likely increased at the time of testing for play behavior. However, in contrast to FAAH inhibition, prior reports indicate that social play behavior is not affected by acute inhibition of MAGL (Fontenot et al. 2018; Manduca et al. 2015). Thus, we speculate that the changes in interactive/physical play behavior observed in the present study were the results of either cumulative effects or adaptations that emerged as a result of repeated/persistent 2-AG elevation. Notably, URB effects were only evident when rats were tested in a novel environment, consistent with prior reports that URB-induced increases in play behavior are only observed when rats are tested in an unfamiliar environment (Manduca et al. 2014). By contrast, JZL-treated rats showed increased play behavior when tested in either a familiar or novel context.

### Adult Social Preference

Adolescent URB, but not JZL, had pronounced effects on social choice in adulthood suggesting a critical role for AEA during adolescence in promoting adult social preference. URB-treated adolescent rats showed heightened preference for a novel partner in adulthood and displayed sustained interest in a familiar partner (vs. a novel object) under conditions where JZL and vehicle-treated rats did not. Interestingly, while the other groups showed preference for a novel object over an empty compartment, URB treated rats were indifferent, showing no object preference under any condition. In humans, AEA has been found to be critical for social processing (Jones et al. 2025). These findings are paralleled by reports in rats that CB1-dependent AEA signaling, driven by oxytocin, promotes social preference (Wei et al. 2015). Considering that brain AEA levels increase during socialization with a novel partner during adolescence (Marco et al. 2011), it is possible that AEA signaling during this phase of development organizes brain circuitry to promote socialization in adulthood. These observations could have relevance to conditions associated with disrupted socialization such as autism spectrum disorder (ASD). Accordingly, it has been reported that URB attenuates social deficits in rat Fmr1-Δexon 8 (Schiavi et al. 2023) and valproic acid (Wu et al. 2020) models of ASD. Interestingly, URB administration during adolescence has also been reported to reverse deficits in social preference following early life adversity in a limited bedding model of resource scarcity (Alteba et al. 2020), although this effect was restricted to late adolescence (PND45–60) (Alteba et al. 2021). Early life stressors have been reported to produce persistent brain region-specific reductions in AEA (Hill et al. 2019; Marco et al. 2013) and URB has been reported to reverse traumatic stress-related social deficits in adult rats (Morena et al. 2018). Thus, URB-induced increases in AEA during adolescence could buffer deficits resulting from early life experiences and restore the developmental effects of AEA that promote adult social behaviors. In contrast to reports that have found that adolescent MAGL inhibition can attenuate deficits in social behaviors following early life stress (Alteba et al. 2020; Fontenot et al. 2018), we found no effects of adolescent JZL on adult social choice. Thus, effects of adolescent 2-AG elevation may require a history of early life stress.

### Adult Cocaine Seeking

Adolescence is a critical period during which life events can influence the risk for SUDs into adulthood. Reports that victimization during adolescence promotes drug use in people (Sullivan et al. 2006) are paralleled by findings that adolescent social isolation (Baarendse et al. 2014; Noschang et al. 2021) and social defeat stress (Burke and Miczek 2014) promote cocaine self-administration (SA) during adulthood in rodents. While the effects of adolescent cannabis use on adult substance use and addiction have been characterized (see e.g., (Ellgren et al. 2007; Friedman et al. 2019; Orihuel et al. 2021), the effects of altered eCB levels have not been well-explored. Here we report heightened cocaine seeking in rats that received JZL during adolescence. While JZL-treated rats did not differ in the acquisition of cocaine SA or initial cocaine SAunder a “low-effort” fixed ratio schedule, when tested under a progressive ratio schedule, responding was increased relative to controls. When rats were retested under the FR schedule, statistically significant treatment effects were once again not observed, although responding tended to remain higher in JZL-treated rats. When tested for drug seeking in the absence of cocaine delivery (i.e., on day one of extinction), responding in JZL rats was markedly higher than in the other groups. Drug seeking did extinguish in JZL-treated rats and, unexpectedly, no effects on drug-primed reinstatement were observed. Altogether, these findings suggest that elevated 2-AG levels during adolescence may heighten the risk for adult drug seeking behavior. While acute increases in 2-AG signaling have been found to promote cocaine-seeking behavior (Engi et al. 2021; McReynolds et al. 2018; Mitra et al. 2021), this is the first report that elevated 2-AG during adolescence may be associated with increased adult cocaine SA and seeking. Interestingly, adolescent URB treatment also produced marked (though not statistically significant) increases in cocaine lever responding, but only upon the initial transition from the FR to PR schedule with no differences from vehicle control rats observed thereafter (i.e., days 3 and 4 of testing). The reason for this observed effect is unclear but could be related to deficits in behavioral flexibility.

### Anxiety-Related Behaviors

We did not observe effects of either JZL or URB treatment during adolescence on behavior on the elevated plus maze in adolescents or open field behavior in adults. This was somewhat unexpected, considering the well-established role for AEA and 2-AG in anxiety-related behaviors (Petrie et al. 2021). However, the findings are consistent with other reports that manipulation of eCB signaling during adolescence does not alter open field or elevated plus maze behavior in adulthood in rats (Biscaia et al. 2003; Cossio et al. 2020; Farinha-Ferreira et al. 2022; Llorente-Berzal et al. 2011; Rubino et al. 2008). Notably, it has been found that while URB during adolescence has no effect on open field behavior alone, it does prevent anxiety-related responses that otherwise emerge as a result of early-life stress (i.e., limited bedding prior to weaning; (Alteba et al. 2021)). FAAH polymorphisms that reduce enzymatic activity and elevate AEA levels have been associated with a lower risk for adolescent-onset anxiety in humans and mice (Desai et al. 2024; Gee et al. 2016; Gerhard et al. 2023) and have been associated with a higher risk of PTSD in adolescents (Marusak et al. 2024). Altogether, these findings suggest that eCB signaling during adolescence by itself does not determine risk for adult anxiety disorders but rather intersects with other factors (e.g., early life stress) to influence anxiety-related outcomes in adulthood.

### Implications for Understanding the Effects of Adolescent THC Exposure

While a large body of evidence indicates that adolescent cannabis use by humans and adolescent THC exposure in rodents produce widespread behavioral and biological effects in adulthood (see e.g., (Ferland et al. 2023), (reviewed in (Bara et al. 2021)), the observed effects of adolescent URB or JZL should not be presumed to align with those of THC. In addition to differences in the temporal and spatial patterns of CB1 receptor engagement related to direct (THC) vs. indirect (URB and JZL) receptor agonism, the effects of THC at CB1 receptors differs from those of 2-AG and AEA in terms of affinity and intrinsic efficacy (2-AG is a full agonist while THC and AEA are partial agonists). Moreover, the non CB1 receptor target profiles differ among THC, 2-AG, and AEA. Most notably, AEA is a potent agonist at TRPV1 (Ross 2003), and the three compounds have been reported to have varying actions at other targets, such as the CB2 receptor (Howlett and Abood 2017), peroxisome proliferator-activated receptor (Iannotti and Vitale 2021), and orphan receptors, including GPR55 (Ross 2009). Nonetheless, there are some similarities between our observed effects of adolescent URB or JZL administration and the effects of adolescent THC reported by others. For example, similar to our finding that adolescent JZL increases cocaine seeking in adulthood, adolescent THC has been reported to increase cocaine self-administration in adulthood (Friedman et al. 2019; Orihuel et al. 2021). Others have reported that adolescent THC also increase adult heroin self-administration (Ellgren et al. 2007). However, in many cases reported effects of adolescent THC differed from those that we observed with JZL and URB. In contrast to the increases in social play behavior that we observed following adolescent JZL delivery, it has been reported that adolescent THC administration decreases social play behaviors (Keeley et al. 2021). We did not observe effects on JZL or URB on elevated plus maze or open field behavior, while others have reported that adolescent THC heightens anxiety-like behaviors assessed using these approaches ((De Gregorio et al. 2020; Renard et al. 2017; Stopponi et al. 2014) but see (Bruijnzeel et al. 2019)). Finally, in contrast to the increased social preference that we observed following adolescent URB treatment, adolescent THC has been reported to produce deficits in social choice (Renard et al. 2017).

### Study Limitations

There are several limitations to the present study. First while doses of URB and JZL administered during adolescence were selected based on prior rodent studies demonstrating increases in AEA (Fegley et al. 2005) and 2-AG (Long et al. 2009; Pan et al. 2009), respectively, we did not measure blood or brain eCBs in the present study. Second, in addition to catabolizing AEA and 2-AG, FAAH and MAGL also break down other lipids which could contribute to drug effects (van Egmond et al. 2021). Pretreatment with CB1 receptor antagonists will be needed to confirm that drug effects involve eCB signaling. Third, the present study examined the effects of elevated AEA and 2-AG during adolescence. Future studies using inhibitors of eCB synthesis (e.g., the DAG lipase inhibitor DO34 and the NAPE-PLD inhibitor LEI-401) are needed to determine the impact of reduced eCBs during adolescence. Fourth, while we observed effects of elevating eCBs during adolescence, we did not test if drug effects were selective for the adolescent period. Moreover, eCB signaling varies within the adolescent window (Ellgren et al. 2007; Lee et al. 2013) and the effects of elevating eCBs during adolescence on adult behavior have been reported to differ during mid- (PND30–45) vs. late- (PND45–60) adolescence (Alteba et al. 2021). Better understanding specific age windows in which eCBs influence behavior will require further investigation. Finally, only male rats were used in the present study. Prior work has demonstrated sex specific effects of URB (Jankovic et al. 2022; Zer-Aviv and Akirav 2016) and JZL (Jacotte-Simancas et al. 2023) and sex-dependent effects of THC administration during adolescence on behavioral and biological endpoints have been reported (Freels et al. 2024; Martinez et al. 2025; Silva et al. 2016). Assessment of sex differences in the effects of adolescent eCB manipulations across the lifespan is an important future research goal.

## Conclusions

These results highlight the importance of adolescent eCB signaling in establishing the trajectory for healthy and unhealthy behaviors into adulthood. As altered eCBs during adolescence have been associated with a number of conditions (Tansey et al. 2025), including depression (Jaster et al. 2025), anxiety disorders (Marusak et al. 2025), autism spectrum disorder (Aran et al. 2019) and PTSD (Marusak et al. 2024), our findings may have implications for early diagnosis and interventions in adolescent populations.

## Supplementary Material

Supplementary Files

This is a list of supplementary files associated with this preprint. Click to download.

• Estesetal2025PsychopharmacologySupplementary.docx

## Figures and Tables

**Table 1:** Effects of adolescent JZL184, URB597, and vehicle administration on body weights. Datarepresent grams (±S.E.) on post-natal day PND30, PND40, and PND65 in rats treated with vehicle (Veh), JZL184 (JZL), or URB597 (URB) during adolescence.

Group	PND30	PND40	PND65
*Veh*	106.72 ±1.70 g	177.22 ±2.71 g	322.89 ±4.11 g
*JZL*	105.83 ±1.77 g	180.11 ±1.79 g	321.11 ±3.47 g
*URB*	103.50 ±1.79 g	180.06 ±2.45 g	319.39 ±4.56 g

## Data Availability

Data will be made available in a publicly accessible repository
